# Application of nanomaterials in treatment, anti-infection and detection of coronaviruses

**DOI:** 10.2217/nnm-2020-0117

**Published:** 2020-05-07

**Authors:** Ghazal Nikaeen, Sepideh Abbaszadeh, Saeed Yousefinejad

**Affiliations:** 1 Research Center for Health Sciences, Institute of Health, School of Health, Shiraz University of Medical Sciences, Shiraz, Iran

**Keywords:** antiviral, bio-labeling, coronaviruses, COVID-19, gold nanoparticles, protein nanoparticles, severe acute respiratory syndrome coronavirus, silver nanoparticles, transmissible gastroenteritis virus, vaccine nanotechnology

## Abstract

Nanotechnology and nanomedicine have excellent potential in dealing with a range of different health problems, including viruses, which are considered to be a serious challenge in the medical field. Application of nanobiotechnology could represent a new avenue for the treatment or disinfection of viruses. There is increasing concern regarding the control of coronaviruses, among these, Middle East respiratory syndrome coronavirus, severe acute respiratory syndrome coronavirus and severe acute respiratory syndrome coronavirus-2 are well known and dangerous examples. This article aims to provide an overview of recent studies on the effectiveness of nanoparticles as diagnostic or antiviral tools against coronaviruses. The possibilities of effectively using nanomaterials as vaccines and nanosensors in this field are also presented.

## Graphical abstract

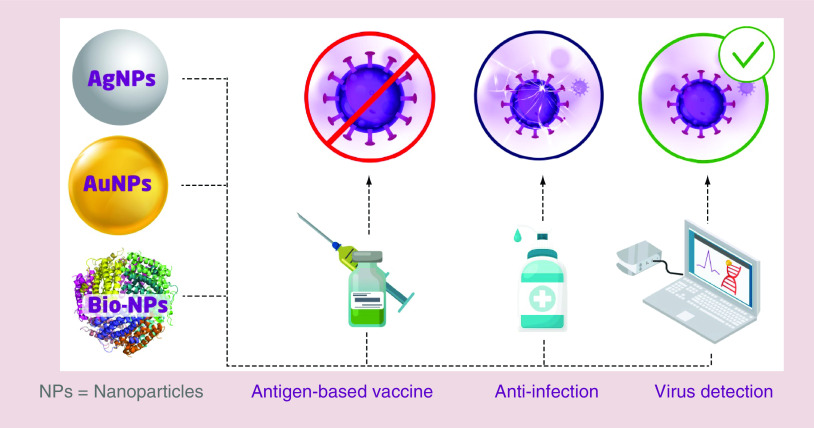

For years, nanotechnology has attracted much attention and has been widely utilized in various fields such as medicine [1], agriculture [2–4] and bio-labeling [5,6]. Nowadays, nanotechnology has been used in many different parts of medical science, such as gene delivery [7], targeted drug delivery [8,9], imaging [10], artificial implants [11] and sensing platforms for public safety and other biosensors [12,13]. Furthermore, it can be exploited for treatment or diagnosis of cancer [14] and development of potent agents against viral, bacterial and fungal infections. The rationale of this interest in using nanotechnology in medicine is the nanometer size of the materials, which allows entrance into the cells of living systems and especially into the human body. In addition, nanomaterials can play a protective role, preventing the encapsulated drug or anti-infection agent from degradation because of the shielding properties of these nano-sized materials [15,16].

Viral infections, due to their problematic wide spreading and their ability to evolve by genetic mutation can pose a great threat to human health. In the last few decades, the high number of deaths caused by some viral infections has been also a challenge for healthcare systems [17]. The Coronaviridae family, along with three other virus families, the Roniviridae, Arteriviridae and Mesoniviridae, are categorized in the Nidovirales order because of their similarity in their distinctive replication strategy and function in hosts [18,19]. The Coronaviridae family, commonly named as coronaviruses (CoVs), is composed of two subfamilies: Coronavirinae and Torovirinae. Infection with a member of the Coronavirinae subfamily can lead to any of reproductive disease, pneumonia, polyserositis, enteritis, hepatitis, encephalomyelitis, nephritis or sialo dacryoadenitis in mammals and birds. Coronavirus and coronavirus-like infections have been reported in various wild and livestock animals such as swine, horses, cattle, cats, dogs, camels, birds and bats [18]. In humans, coronaviruses include a wide range of viruses contributing to the common cold as well as to more severe respiratory diseases such as Middle East respiratory syndrome (MERS), severe acute respiratory syndrome (SARS) and novel coronavirus. Since coronaviruses can be transmitted between animals and people, their effect is known as zoonotic diseases. Previous studies have found that MERS and SARS coronaviruses jumped to humans from dromedary camels and civet cats, respectively, before being transmitted to humans. There are various known coronaviruses which can infect animals that have not been seen in humans yet [20].

All recent epidemics of coronavirus have emerged unexpectedly and can spread easily. They not only threaten human health but also may lead to catastrophic consequences [21]. In 2003, the SARS-CoV epidemic occurred. More than 8000 people were infected, leading to about 900 deaths worldwide [22]. MERS-CoV was first identified in 2012 and according to a report from June 2019, 2494 individuals have been infected with MERS-CoV since 2012, with a death rate of 34.4% [23]. In late 2019 and early 2020, a number of human cases of new coronavirus infection were reported. It has been suggested that the virus arose in relation with the Huanan Seafood Wholesale Market in Wuhan, China; however, at the time of writing, there has been no confirmation of where the virus originated. The novel coronavirus was confirmed by China on 7 January 2020 and was later named as ‘SARS-CoV-2’, which causes the novel coronavirus disease. On 11 March 2020, the WHO declared the novel coronavirus outbreak as a pandemic. On 23 April 2020, a total of 2,510,177 cases had been confirmed in worldwide and 172,241 deaths reported [24].

Depending on the seriousness of the disease and the mode of transmission, appropriate methods are required to minimize the transmission of infectious diseases [25]. For example, some diseases are entirely preventable by vaccination (e.g., measles and polio) or by access to improved sanitation (e.g., diarrheal and parasitic diseases); and others are treatable when detected in a timely manner (e.g., tuberculosis and malaria). To date, there is no specific treatment or vaccine found to be successful in the treatment of individuals infected with SARS-CoV-2 [26].

Due to high rate of mortality and rapid global transmission of coronaviruses, this article aims to review the use of nano-sized materials for treatment, detection and antiviral activity against previous known coronaviruses. These may present a way to proceed for application of nanotechnology against new coronaviruses. In this special report, different strategies of using nanoparticles in dealing with coronaviruses are discussed in three parts: applications in nano-based vaccines, antiviral activity and development of diagnostic sensors.

## Nano-based vaccine candidates & treatments for coronaviruses

Vaccination has been known as one of the most effective medical intervention used to stimulate immune response against infectious disease [27]. Meanwhile, as nanoparticles have been proven to have immunostimulatory effects [28], a great deal of attention has been given to development of nano-based therapeutic agent or vaccines against different types of coronaviruses.

For example, in 2011, Staroverov *et al.* evaluated the protective immune response stimulated by the administration of gold nanoparticles (Au NPs) conjugated with a type of coronavirus known as swine transmissible gastroenteritis virus (TGEV) in immunized mice and rabbits [29]. TGEV-conjugated colloidal gold was found to elicit higher concentrations of IFN-γ and superior titers of neutralizing antibody in vaccinated animals. Immunization with the antigen-colloidal gold complex increased propagation of T cells tenfold in comparison with the response to free antigen and the authors of the study also reported that administration of the complex resulted in subsequent activation of macrophage respiratory activity and higher protective immunity against TGEV. Thus, AuNPs conjugated to a virus could be considered as a potential antiviral candidate for vaccine application.

Kim *et al.* proposed a ferritin-based NP assembly mediated by RNA as a novel molecular chaperone and demonstrated that using their NP-based vaccine against MERS-CoV can induce CD4^+^ T cells, which in turn leads to the generation of IFN-γ and TNF-α upon antigen stimulation [30]. Additionally, Jung *et al.* attempted to develop an immunogenic vaccine against MERS-CoV using a heterologous prime-boost strategy involving a recombinant adenovirus serotype 5 encoding the MERS-CoV spike gene (Ad5/MERS) and spike protein nanoparticles [31]. Groups of female BALB/c mice were immunized three times with the prime-boost vaccination. It was shown that the homologous spike protein NPs successfully induced higher antibody titers compared with the Ad5/MERS only group. However, a Th1 immune response was not observed to be provided by spike protein NPs themselves and only a Th2 immune response involving induction of neutralizing antibodies was elicited. Therefore, in order to provide much more durable immunogenicity and a suitable balance of Th1/Th2 responses, a heterologous one-stage Ad5/MERS prime and two-stage spike protein NPs boost seemed to be more effective than the homologous prime-boost regimen using either Ad5/MERS or spike protein nanoparticles alone.

**Figure 1.**
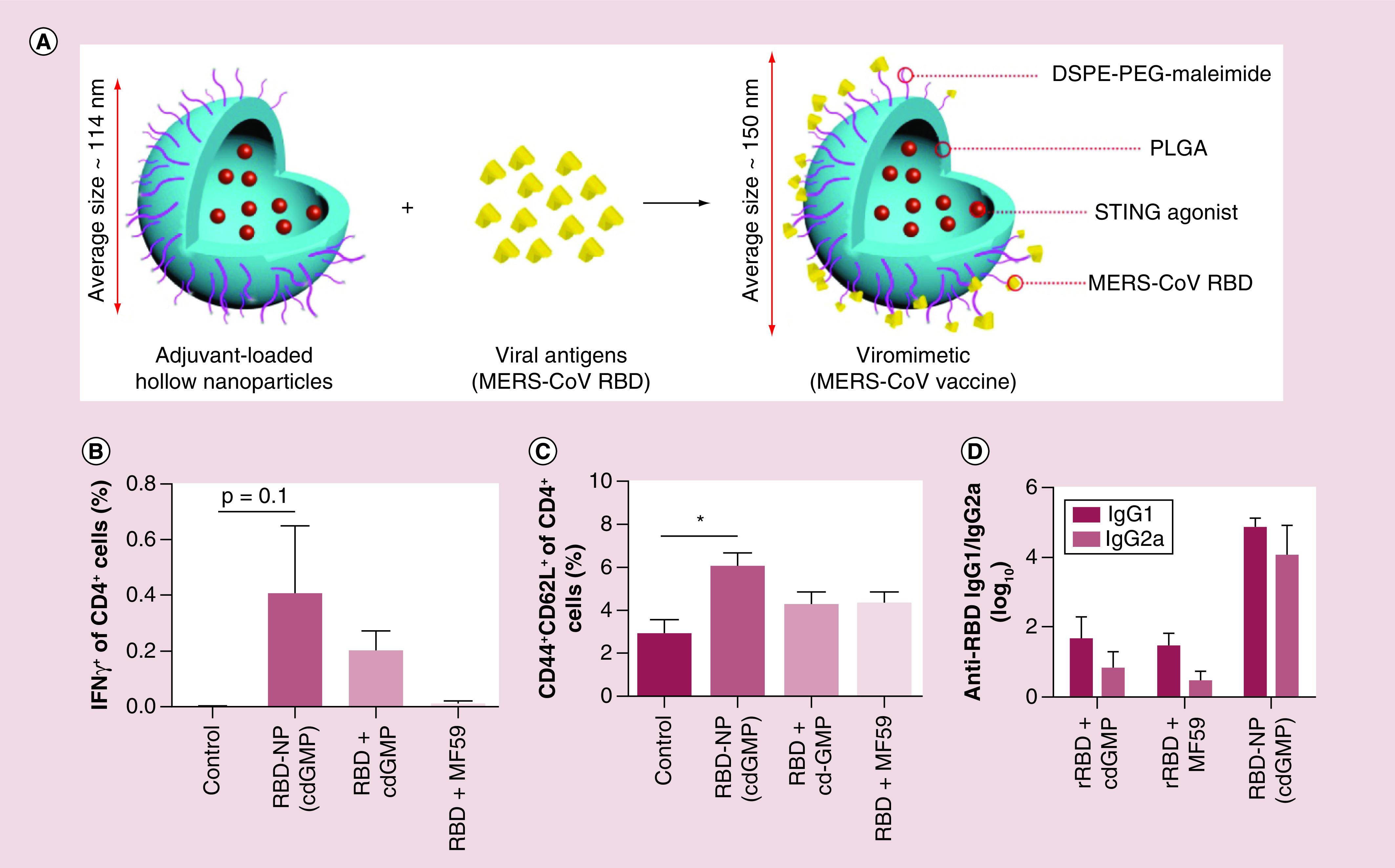
Preparation of viromimetic nanoparticle vaccine and robust humoral and CD4+T cell responses. **(A)** Schematic representation for the preparation of viromimetic nanoparticle vaccine. Hollow PLGA nanoparticles with encapsulated adjuvant and surface maleimide linkers were prepared using a double emulsion technique. Recombinant viral antigens were then conjugated to the surface of nanoparticles via thiol-maleimide linkage. **(B)** CD4^+^ T-cell responses against MERS-CoV RBD in immunized mice were determined by intracellular cytokine staining on day 7 after boost, with n = 3 to obtain error bars. **(C)** Frequencies of central memory (CD44^+^CD62L^+^) CD4^+^ T cell in the draining lymph nodes of immunized mice 28 days after boosting with n = 3 to obtain error bars. Statistical analyses were performed by unpaired *t*-tests (*p < 0.05). **(D)** MERS-CoV RBD-specific IgG1 and IgG2a titers in immunized mice on day 35 postvaccination with n = 6 to obtain error bars. CoV: Coronavirus; MERS: Middle East respiratory syndrome; PLGA: Poly(lactide-coglycolide); RBD: Receptor-binding domain. Reproduced with permission from [32] © Wiley-VCH Verlag GmbH & Co. KGaA (2019).

In 2019 Lin *et al.* developed a novel viromimetic nanoparticle-based vaccine coupled with an immunologic stimulator of interferon genes agonist adjuvant against MERS-CoV [32]. As shown in Figure 1, a hollow polymeric nanocarriers coated with receptor binding domain (RBD) antigens were prepared followed by loading with cyclic diguanylate monophosphate as an emerging class of stimulator of interferon genes agonist adjuvant. C57BL/6 mice were then immunized with the developed vaccine. Lin *et al*. reported that the virus-like NPs induced durable cellular and humoral immune responses. A strong and constant humoral and CD4^+^ T-cell response was also detected in the studied mice immunized with the virus-like NP vaccine compared with free RBD antigen admixed with either free cyclic diguanylate monophosphate or MF59 (AddaVax), an adjuvant for influenza vaccines that has been utilized clinically (see Figure 1B–C). In contrast to MF59, this viromimetic NP vaccine induced higher levels of humoral responses. Furthermore, mice immunized with the proposed NP vaccine produced high levels of RBD-specific IgG2a antibodies without induction of lung eosinophilic immunopathology after the infection (See Figure 1D). In a recent study performed by Sekimukai *et al.*, the efficacy of two types of adjuvants (AuNPs and Toll-like receptor agonists) with recombinant S protein were evaluated against SARS‐CoV infection in mice [33]. Results indicated that vaccination with AuNP‐adjuvanted protein elicited strong IgG response but, in contrast to a Toll-like receptor agonist‐adjuvanted vaccine, did not result in induction of protective antibodies and decreasing eosinophilic infiltration.

Thus, owing to their immunogenic properties, various types of NPs, including gold NPs, spike protein NPs and hollow polymeric NPs have all been reported to have considerable potency to induce an immune response against coronaviruses in animal models and *in vitro*. Different application of NPs-based vaccine candidates with some details regarding function and mechanism are summarized in Table 1.

**Table 1.** Application of nanoparticles-based vaccine candidates against coronaviruses with some details regarding function and mechanism.

NPs type (carrier)	Type of coronavirus (antigen)	Adjuvant	Target life system	Antiviral mechanism	Disadvantage	Ref.
Au NPs	Swine TGEV	Au NPs	Mice and rabbits	Increase of the peritoneal macrophages respiratory activity and plasma IFN-γ level		[29]
Ferritin-based NPs	MERS-CoV (RBD antigen)	–	BALB/c mice	Induction of CD4^+^ T cells and IFN-γ TNF-α responses	–	[30]
Spike protein NPs	MERS-CoV	Aluminum	BALB/c mice	Induction of higher titers of neutralizing antibody and Th2 immune response without induction of Th1 immune response.	Imbalance of Th1/Th2 responses and short-term protection immunity.	[31]
Hollow polymeric NPs	MERS-CoV (RBD antigen)	STING agonist (cdGMP)	C57BL/6 mice	Induction of higher levels of humoral responses and IgG2a antibodies without induction of lung eosinophilic immunopathology	–	[32]
Au NPs	SARS‐CoV	Au NPs	BALB/c mice	Induction of IgG response	Fail to reduce eosinophilic infiltration	[33]

cdGMP: Cyclic diguanylate monophosphate; CoV: Coronavirus; MERS: Middle East respiratory syndrome; NP: Nanoparticle; RBD: Receptor-binding domain; SARS: Severe acute respiratory syndrome; STING: Stimulator of interferon genes; TGEV: Transmissible gastroenteritis.

## Antiviral activity

Nanomaterials have regularly been applied in as antiviral agents [34,35] or as delivery platforms for antiviral compounds [36]. This section assesses the application of nanomaterials in developing antiviral agents against certain coronaviruses.

In a patent invented in 2014 by Cho *et al.*, a mixture comprising silver colloid, titanium dioxide (TiO_2_) NPs, a dispersion stabilizer, a binder and water showed its antibacterial, antifungal and antiviral activities (US 8,673,331 B2) [37]. Results of antiviral tests showed that at 100-fold dilution of the composition concentration showed an antiviral activity against porcine epidemic diarrhea virus (PEDV) and TGEV at a rate of 99.99% or higher. On the other hand, when the composition concentration was 1000-fold diluted, the inhibited growth of the viruses was at a rate of 99.9% for PEDV and 93.0% inhibition for TGEV. The antiviral activity of the nanomaterial proposed Cho *et al.* was therefore dependent on composition concentration meaning dosage should be adjusted to have desired inhibition [37].

In 2014, Lv *et al*. compared the strength of immune responses induced by four different silver nanomaterials, including silver NPs, silver nanowires of 60 and 400 nm and silver colloids, on TGEV in infected swine testicle cells [38]. At a concentration of 3.125–12.5 μg/ml, the percentage reduction in virus titer was evaluated in different silver nanomaterials. It was shown that of the different types of silver nanomaterials, only Ag NPs and the two types of Ag nanowires induced protection against TGEV. The silver colloids were not reported to restrain cellular entry of the virus. The Ag NPs and silver nanowires were capable of reducing the number of apoptotic cells elicited by TGEV.

In the year 2017, Hu *et al.* developed a promising treatment approach based on the nanoformulation of diphyllin for the treatment of feline infectious peritonitis (FIP), which is caused by feline coronavirus [39]. Diphyllin is a vacuolar ATPase that has been demonstrated to inhibit endosomal acidification in fcwf-4 cells, a necessary process for virus uncoating and cellular entry. It was shown that poly(ethylene glycol)-block-poly(lactide-coglycolide), which was used as the diphyllin nanocarrier, enhanced the inhibitory activity of diphyllin against FIP and also improved the safety profile. The antiviral activity of diphyllin nanoparticles was also investigated. It should be noted that administration of high doses of the nanoparticles were found to be tolerable in mice. Therefore, diphyllin nanoparticles proved to have prominent antiviral effect against FIP. While not studied as a vaccine candidate, this study still demonstrates that nanoformulations can be effective against coronaviruses and this specific example could be a potential treatment candidate.

A novel therapeutic strategy based on Ag nanomaterials against the alphacoronavirus PEDV was first introduced in 2018 by Du *et al.* [40]. They demonstrated that Ag_2_S nanoclusters (NCs) could restrain PEDV proliferation in treated Vero cells. As suggested by authors, this may be attributed to the fact that treatment with Ag_2_S NCs inhibited the viral budding and the synthesis of viral negative-strand RNA. Further, the Ag_2_S NCs were found to positively regulate the proliferation of IFN-stimulating genes and the expression of pro-inflammation cytokines, leading to protection against PEDV infection, making them a good option to be used in further studies as a treatment tool.

**Figure 2.**
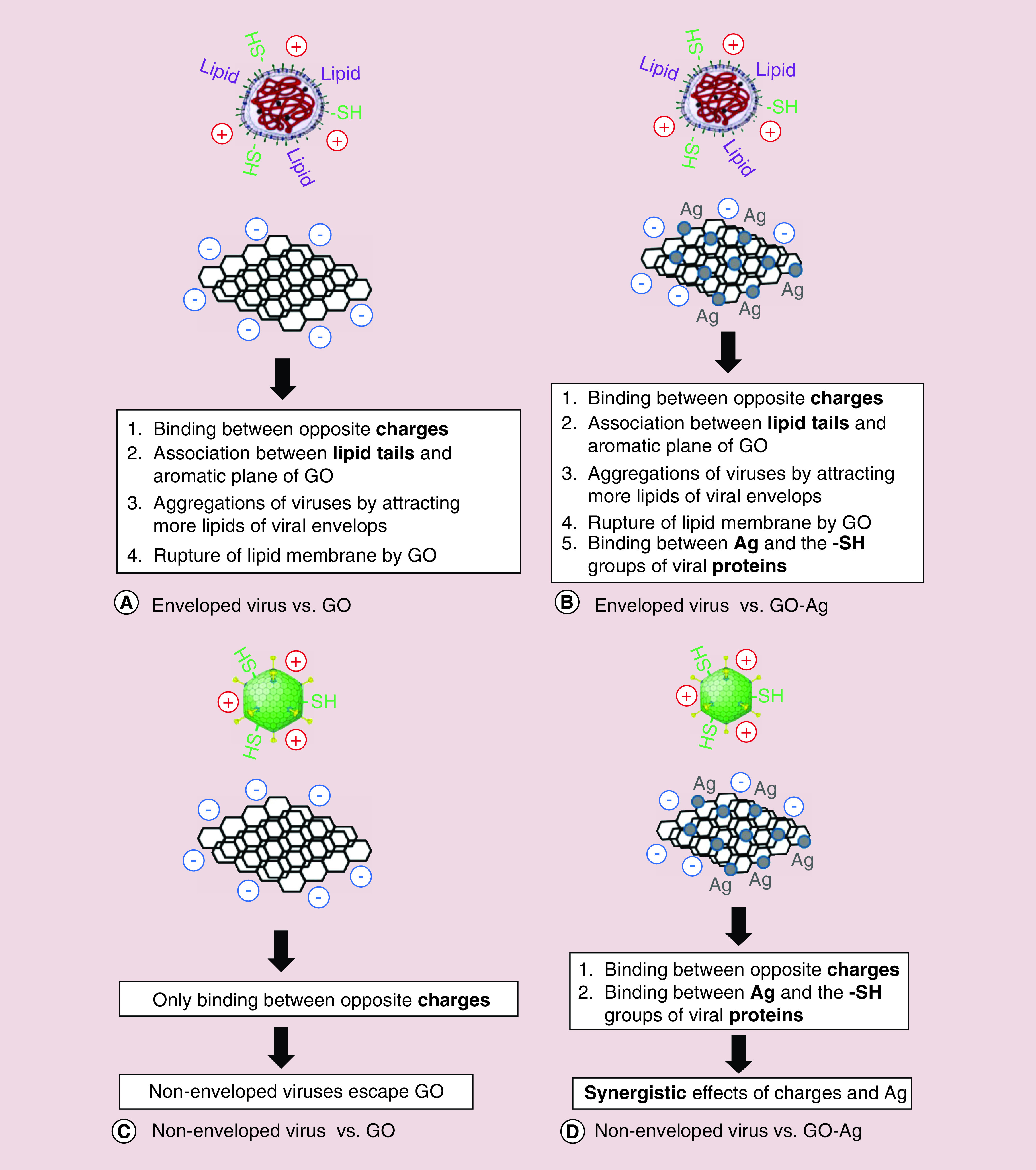
Schematic for the antiviral mechanisms of studied graphene and Ag nanomaterials. **(A)** GO against the enveloped virus; **(B)** GO-Ag against the enveloped virus; **(C)** GO against the nonenveloped virus; **(D)** GO-Ag against the nonenveloped virus. GO: Graphene oxide; GO-Ag: Graphene-silver nanocomposite. Reproduced with permission from [41], licensed with CC-BY 4.0.

The antiviral activity of graphene oxide–silver (GO–Ag) nanocomposites was reported against non-enveloped and enveloped viruses by Chen *et al.* in 2016 [41]. To evaluate the antiviral activity of GO-Ag, GO–Ag solutions with different dilution orders were incubated with serially diluted solution of feline coronavirus. After removing the composite particles, the supernatant was tested using a virus inhibition assay. They demonstrated that 0.1 mg/ml of GO–Ag can inhibits 24.8% of feline coronavirus infection. GO–Ag showed higher efficacy when compared with treatment with GO alone. As illustrated in Figure 2, during the process of restraining viral infection, GO sheets, which were negatively charged, interact with the positively charged lipid membranes and silver particles bind to the sulfur groups of the viral proteins. As a result, it has been shown that GO could only have an inhibitory effect on enveloped viruses at noncytotoxic concentrations while GO sheets containing AgNPs are capable of impeding the infection caused by both nonenveloped and enveloped viruses.

To tackle human coronavirus NL63, Ciejka *et al.* developed a biopolymeric material to form nano/microspheres (NS/MS) which had good potential for adsorbing coronaviruses [41]. The investigation demonstrated that after addition of N-(2-hydroxypropyl)-3-trimethyl chitosan (H-HTCC)-NS/MS to viral suspensions there was a decrease in the copy number of viral RNA, this showed good correlation with the amount of added H-HTCC-NS/MS. Their study showed that 2.5 mg/500 μl H-HTCC-NS/MS could cause a decrease equal to 99.60%. When 10 mg/500 μl of HHTCC-NS/MS was added, there was a decrease of 99.92%.

Therefore, nanomaterials can have antiviral applications against a range of coronaviruses. However, more focus needs to be placed in investigating antiviral nanomedicines against SARS-CoV, MERS-CoV and SARS-CoV-2. A summary about the applied NPs-based antiviral agents for disinfection of coronaviruses with some details on their function and inhibition efficiency are represented in Table 2.

**Table 2.** Application of nanoparticles-based antiviral agents for disinfection of coronaviruses with some details regarding function and inhibition efficiency.

Virus	Host	Antiviral substances	Description of antiviral activity type	Antiviral substances concentration	Inhibition	Ref.
FCoV	Cat	GO-Ag nanocomposite	Antiviral activity against enveloped viruses	0.1 mg/ml	24.80%	[41]
PEDV	Pig	Composites with silver colloid and titanium dioxide nanoparticles	Inhibited the growth of the viruses	1000-fold diluted from original	≥99.90%	[37]
PEDV	Pig	Ag_2_S nanoclusters	Inhibition of viral proliferation (*in vitro* cells = Vero)	46 μg/ml	≥99.9%	[40]
TGEV	Pig	Composites with silver colloid and titanium dioxide nanoparticles	Inhibited the growth of the viruses	1000-fold diluted from original	≥93%	[37]
TGEV	Pig	Ag NPs, Ag NW	Inhibition of cell apoptosis induced by the virus (*in vitro* cells = ST cells)	3.125–12.5 μg/ml	7.05–67.35%	[38]
FIPV	Cat	Diphyllin nanoparticles	Inhibition of endosomal acidification responsible for virus uncoating and cytoplasmic entry (*in vitro* cells = fcwf-4)	2 μM	≥99.9%	[39]
HCoV-NL63	Human	Bio polymeric nano/microspheres (HTCC-NS/MS)	Capable of adsorbing coronaviruses	10 mg/500 μl	99.92%	[42]

CoV: Coronavirus; FCoV: Feline coronavirus; GO-Ag: Graphene-silver nanocomposites; HTCC-NS/MS: N-(2-hydroxypropyl)-3-trimethyl chitosan-nano/microsphere; MERS: Middle East respiratory syndrome; NP: Nanoparticle; NW: Nanowire; PEDV: porcine epidemic diarrhea virus; ST: Swine testicle; TGEV: Transmissible gastroenteritis.

## Nanosensors for diagnosing coronaviruses

Detecting variations in DNA sequences has been found to have a key role in diagnosing and subsequently treating genetic-related conditions at early stages. Among the various types of genetic alteration diseases, sequence-specific mismatch is of significant importance; however, detection of this variation is very difficult and this problem is more serious in case of single-nucleotide polymorphism [43]. It is noteworthy that sequence-specific detection is an important and interesting topic in different medical fields among which pathogen response studies, inherited diseases diagnosis and bacterial/viral detection can be highlighted [44].

In the year 2017, Teengam *et al*. developed a multiplex colorimetric paper-based analytical device using Ag NPs as a colorimetric reagent for the detection of DNA associated with viral infection such as MERS-CoV. In their study, under an optimum condition, a limit of detection of 1.53 nM was achieved [45]. Another study, performed by Layqah and Eissa in 2019, described an electrochemical immunosensor utilizing an array of carbon electrodes which were modified with Au NPs which enabled detection of human coronavirus (HCoV) and MERS-CoV proteins in spiked nasal samples [46]. The method was conducted with lowest detection limit of 1 and 0.4 pgml^-1^ for MERS-CoV and HCoV in 20 min, respectively, and a good linear relationship were observed between the sensor response and virus concentrations. Linear ranges of 0.001–100 ngml^-1^ and 0.01–10,000 ngml^-1^ were obtained for MERS-CoV and HCoV, respectively.

**Figure 3.**
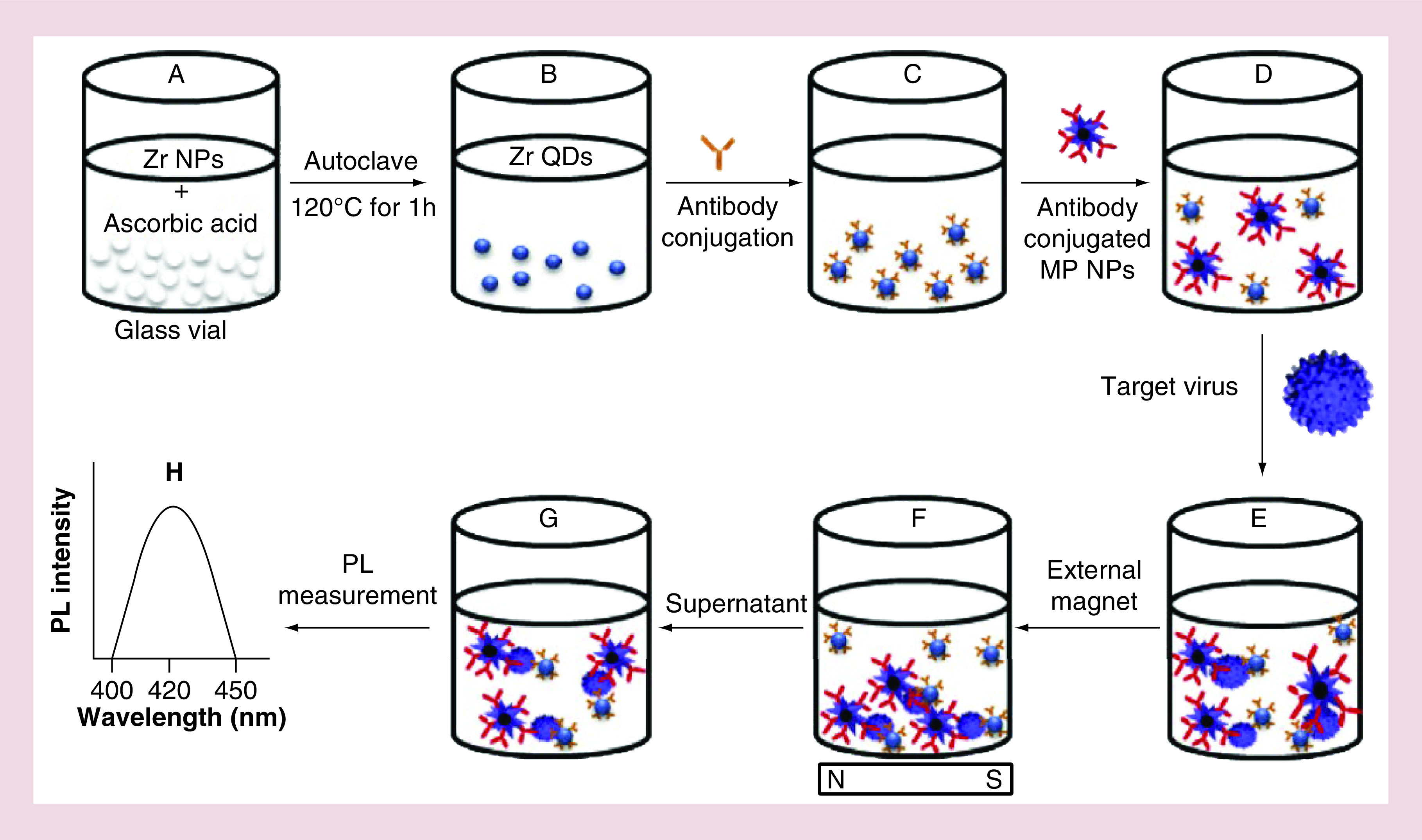
Schematic representation of virus sensor design based on Zr nanomaterials. **(A)** Zr NPs and reducing agent kept in vial; **(B)** Zr QDs formation; **(C)** antibody conjugated QDs; **(D)** the addition of antibody-conjugated MP NPs; **(E)** formation of nanostructured magnetoplasmonic-fluorescence with the addition of target virus, then separated **(F)**; **(G)** the nanohybrid-conjugated part was dispersed and the optical properties measured **(H)**. MP: Magnetoplasmonic; NP: Nanoparticle; QD: Quantum dot. Reproduced with permission from [48], licensed with CC BY-NC-ND 4.0.

A self-assembled nanostructure of chiral gold NPs (CAu NPs) and quantum dots (QDs) was created by Ahmed *et al.* in 2017 and then applied as a chiro-immunosensor for the detection of infectious bronchitis virus (IBV) in chicken blood samples. The presented biosensor proved to be efficient in selective determination of the target virus with a detection limit of 47.91 EID/50 ml egg infective dose (EID) [47]. In another study, Ahmed and coworkers reported an approach for IBV detection by using zirconium quantum dots (ZrQDs) and magnetoplasmonic (MP) NPs [48]. As indicated in Figure 3, after preparation of ZrQDs and MP NPs, the IBV antibodies were conjugated with both nanomaterials. At this step, no attraction or conjugation was observed between the utilized nanomaterials (i.e., ZrQDs and MP) and they were apart from each other till the time of adding the target virus. By addition of a virus, a magnetoplasmonic-fluorescent nanohybrid structure was formed and then separated using an external magnet. Eventually, the concentration of the virus was measured through photoluminescence intensity of the nanohybrids with a detection limit of 79.15 EID/50 ml. In a study by Weng and Neethirajan, an immuno biosensor based on antibody-functionalized MoS 2 for rapid detection of IBV with a limit of detection equal to 4.6 × 10^2^ EID_50_ per ml was developed [49]. In contrast to traditional tests, their immunosensor provided remarkable advantages such as higher sensitivity and lower analysis time, as well as acceptable linearity and validation using the ELISA technique.

In another study, Wang *et al.* developed a nano-nest PCR assay based on AuNPs for discrimination of the variant and classical strains of PEDV [50]. A total of 78 clinical samples collected from different areas in China were assessed by using both nano-nest and common reverse transcription polymerasechain reaction (RT-PCR). Results indicated that the nano-nest PCR assay was more powerful, with 100-fold sensitivity, than common PCR. In order to determine the specificity of this nano-based assay, five other viruses; classical swine fever virus, porcine rotavirus, pseudorabies virus, TGEV and porcine reproductive respiratory syndrome virus were also evaluated. Interestingly, it was shown that this assay did not amplify the DNA or cDNA of the other viruses or even of the classical PEDV strain, leading to the fact that the nano-nest PCR assay can be successfully applied for specific detection of variant PEDV [50].

In a study by Liu *et al.*, an immunochromatographic strip (ICS) test was developed based on five IBV-specific monoclonal antibodies against the target antigens, spike (S) glycoprotein and nucleocapsid proteins (N), for the detection of IBV in infected chickens. During preparation of the ICS, monoclonal antibody–colloidal gold conjugate was used as a tracer in conjugation pad of the strip [51]. According to the results, the best detection limit of 10 ^4.4^ EID_50_ was achieved for IBVs. Moreover, with RT-PCR as a reference, the assembled ICS were identified to be specific to IBV antigens only, compared with other respiratory pathogens including infections laryngotracheitis virus, avian influenza virus and Newcastle disease virus. There was good agreement between the results obtained by the ICS and RT-PCR. Therefore, given the fact that the RT-PCR is an expensive, technically demanding approach, the gold nanoparticles-based ICS technique appeared to have the potential of on-farm rapid detection of various IBV strains in chickens.

The above studies on the recent applications of NPs in developing sensors based on different analytical techniques for coronaviruses and their related limit of detections are compared in Table 3. As it was discussed above AgNPs, MoS_2_ nanosheets, QD-MP NPs, Zr NPs and Au NPs had been applied in detection of a range of coronaviruses. Thus, coupling nanomaterials with colorimetric sensing, electrochemiluminescence, immunosensing, photoluminescence and chiroimmunosensing are potential techniques to detect coronaviruses. In addition, electrochemical devices can also be a good options for detection of new kinds coronaviruses in future because of their good ability for coupling with nanomaterials. The advantages of using nanomaterials in this aspect can decrease the analysis time and also increasing the sensitivity, which can open a new door for designing better approaches and higher performance in future.

**Table 3.** Recent applications of nanoparticles in developing coronaviruses sensors based on different analytical techniques and related limit of detections.

Virus	NPs	Detection way	LOD	Ref.
MERS-CoV	Ag NPs	Colorimetric	1.53 nM	[45]
MERS-CoV	Au NPs	Electrochemiluminescence	1.0 pg.ml^-1^	[46]
HCoV	Au NPs	Electrochemiluminescence	0.4 pg.ml^-1^	[46]
IBV	MoS_2_ nanosheets	Immunosensing	4.6 × 10^2^ EID_50_ per ml	[49]
IBV	QD-MP NPs and Zr NPs	Photoluminescence	79.15 EID/50 ml	[48]
IBV	CAu NPs	Chiroimmunosensing	47.91 EID/50 ml	[47]
PEDV	Au NPs	Nano-nest PCR	2.21 × 10^-7^ ngμl^-1^	[50]
IBV	Colloidal Au NPs	ICS	10^4.4^ EID/50 ml	[51]

CAu NP: Chiral gold nanohybrid; CoV: Coronavirus; HCoV: Human coronavirus; IBV: Infectious bronchitis virus; ICS: Immunochromatographic strip; LOD: Limit of detection; MERS: Middle East respiratory syndrome; NP: Nanoparticle; PEDV: Porcine epidemic diarrhea virus; QD-MP: Quantum dot-magnetoplasmonic.

## Conclusion

Today, there is increasing concern on how to battle coronaviruses as they have been changing the way we live with novel coronavirus disease being a particularly problematic example. Despite considerable efforts which have been put in to developing an effective therapeutic strategy against different types of coronaviruses, no specific approach has yet been identified. Several studies have been conducted on the application of nanomaterials in the treatment, anti-infection and detection of some types of coronavirus, including those discussed in this report, as well as others [52–55]. Thus, an overview of studies regarding the effectiveness of nanoparticles for diagnostic and therapeutic purposes was presented envisioning the possibility of applying nanomaterials in development of a highly effective vaccine against coronaviruses. Up to now, gold, silver, silver sulfide, titanium oxide, zirconium, graphene and some biopolymeric compounds have been the most applicable nanomaterials for this goal. It has been suggested that nanoparticle-based vaccines have a great potential to induce higher protective immunity response in contrast to conventional antigen-based vaccines. Moreover, results have indicated that nano-assays hold promise for providing superior specificity and sensitivity, compared with the current techniques, when applied for rapid detection of viral infection at early stages.

## Future perspective

The quest for finding a reliable, safe and effective vaccine against the SARS-CoV-2 is still underway. Several candidates have been developed ever since its appearance. But at the time of writing this article, there is still no licensed vaccine that can fight it off. According to the previous data obtained from other types of coronaviruses, nano-based vaccines have proven to evoke a more potent immune response. Therefore, further research is recommended to address the administration of nanoparticles toward the study of SARS-CoV-2 in order to create an experimental nano-based-vaccine in a suitable animal model hopefully to provide long-term immunization. Application of nano assays for detection of SARS-CoV and SARS-CoV-2 is also a recommended topic for researchers in this area. Colorimetric sensing, electrochemiluminescence, immunosensing, photoluminescence and chiroimmunosensing and electrochemical sensors are potential techniques to detect coronaviruses in future with the aid of.

**Executive summary**

**Nano-based vaccine candidates & treatments for coronaviruses**

- The field of nanomedicine could be successfully applied for development of the most promising candidates against the coronaviruses.

**Antiviral activity**

- Ag nanoparticles and their compositions can be promising candidate for research on antiviral agents against coronaviruses.
- Most suggested responses in antiviral activity against coronaviruses are concentration dependent.

**Nanosensors for diagnosing coronaviruses**

- Using nanomaterials in detection of coronaviruses has some advantages such as improving sensitivity, detection limit, and the analysis time.
- Nanoparticles can be used for colorimetric detection of coronavirus which is a simple, rapid and low-cost method.
- Gold nanoparticles are good options for immunochromatographic strip test to use in future studies on detection of coronaviruses.
- Electrochemical devices can also be a good options for detection of new kinds coronaviruses in future because of their good ability for coupling with nanomaterials.
